# Caring for caregivers: the impact of the COVID-19 pandemic on those responsible for children and adolescents with type 1 diabetes

**DOI:** 10.1038/s41598-021-85874-3

**Published:** 2021-03-24

**Authors:** Janine Alessi, Giovana Berger de Oliveira, Gabriela Feiden, Beatriz D. Schaan, Gabriela Heiden Telo

**Affiliations:** 1 Postgraduate Program in Medical Science: Endocrinology, Universidade Federal do Rio Grande do Sul, Rua Ramiro Barcelos, 2350, Prédio 12, 4° Andar, Porto Alegre, RS 90035-003 Brazil; 2 Internal Medicine Department, Hospital São Lucas - Pontifícia Universidade Católica Do Rio Grande Do Sul, Porto Alegre, Brazil; 3 School of Medicine, Pontifícia Universidade Católica do Rio Grande do Sul, Av. Ipiranga, 6681 - Partenon, Porto Alegre, RS 90160-092 Brazil; 4 Postgraduate Program in Epidemiology, Universidade Federal do Rio Grande do Sul, Rua Ramiro Barcelos, 2350, Prédio 12, 4° Andar, Porto Alegre, RS 90035-003 Brazil; 5 Associação de Apoio aos Diabéticos do Rio Grande do Sul (AADIRS), Porto Alegre, Brazil; 6 Endocrinology Division, Hospital de Clínicas de Porto Alegre, Porto Alegre, Brazil; 7 National Institute of Science and Technology for Health Technology Assessment (IATS) – CNPq/Brazil, Porto Alegre, Brazil; 8 Graduate Program in Medicine and Health Sciences, Pontifícia Universidade Católica do Rio Grande do Sul, Escola de Medicina da PUCRS, Av. Ipiranga, 6681 - Partenon, 90160-092 Porto Alegre, RS Brasil

**Keywords:** Epidemiology, Human behaviour, Type 1 diabetes

## Abstract

This study aimed to assess the psychological impact of the COVID-19 pandemic on guardians of children and adolescents with type 1 diabetes. An online survey was performed to assess the prevalence of pandemic-related emotional burden, mental health disorders and diabetes-specific emotional burden related to diabetes care during the COVID-19 pandemic. Caregivers of children and adolescents with diabetes under the age of 18 and caregivers of youth without diabetes for the non-diabetes group were invited to participate. For the primary outcome, mental health disorders were evaluated using the Self-Reporting Questionnaire (SRQ-20), while pandemic-related emotional burden and diabetes-specific emotional burden related to diabetes care were evaluated in different domains with specific questions. For analyses, a hierarchical testing strategy was performed. A total of 764 participants were included in the study. Regarding the pandemic period, caregivers of youth with type 1 diabetes endorsed significantly more pandemic-related emotional burden for both themselves (OR 1.67; 95% CI, 1.10 to 2.53) and for their child (OR 2.28; 95% CI, 1.54 to 3.38) when compared to the non-diabetes group. The emotional burden evaluation on different age ranges showed that the two groups were similar when the dependent youth was younger than 6 years. Moreover, a positive screening for mental health disorders during social distancing was higher in the diabetes group compared to the non-diabetes group (OR 2.43; 95% CI, 1.70 to 3.47), particularly in those aged under 12 years old. There was no difference between groups in mental health disorders among caregivers of adolescents older than 12 years. Our results allow to conclude that concern, burden and mental health disorders can be present in caregivers of youth with diabetes, and behavioral changes during the COVID-19 pandemic may enhance this situation.

## Introduction

Caring for children and adolescents with type 1 diabetes mellitus involves daily challenges and can put a psychological strain on those responsible. A study carried out with children under 7 years old shows that caregivers play a fundamental role in controlling the disease and experience the responsibility for its short and long-term consequences, such as episodes of hypoglycemia, future complications, and the impact the disease will have on their children's quality of life^1^. Another study with children under 8 years old shows that, in addition to the responsibility for optimal glycemic control, parents also suffer from constant concerns about the care for their children while the parents are not present. The difficulty in finding someone reliable and used to the demands of diabetes is also challenging. This makes the role of caregiver a full-time job^2^. Despite these issues, concerns tend to be child-centered and the psychological demands of family members involved in childcare are often neglected.

The emotional burden experienced by caregivers of children and adolescents with type 1 diabetes may be aggravated by the current COVID-19 pandemic. The change in family dynamics that has occurred since the beginning of COVID-19 is reflected in different aspects of the care of children with diabetes. First, the concern and fear of a potentially serious infection for the child, constitutes a stressful situation^3^. Second, the suspension of school and extracurricular activities results in more time for children and adolescents at home, requiring greater attention from caregivers. Third, the closing of parks and recreation rooms results in more sedentary habits and, consequently, lower daily energy expenditure^4^. All these changes may significantly impact the glycemic control of the child, adding even more responsibilities and enhancing the stress on guardians.

Despite everything mentioned above, the psychological repercussion of the current scenario in guardians of children and adolescents with diabetes remains hypothetical. Several studies have shown that the deleterious effects of the caregiver’s psychological distress apply to both caregivers and children, resulting in worse glycemic control and increasing the incidence of depression during adolescence^2,5–10^. The care for caregivers’ mental well-being, however, has received little priority when assessing the impact of stressful situations, such as the current pandemic of COVID-19 and the social distancing it requires^11^. The present study aims to assess the psychological impact of routine changes and the demands of the disease on caregivers of children and adolescents with type 1 diabetes during the COVID-19 pandemic, and to identify factors that can guide strategies in similar situations in the future.

## Methods

### Study design and setting

This was a cross-sectional study carried out to evaluate the prevalence of mental health disorders and pandemic-related emotional burden during the COVID-19 pandemic in caregivers of children and adolescents with type 1 diabetes. Caregivers of children and adolescents with a previous diagnosis of type 1 diabetes were recruited to participate in this study as the diabetes group, while caregivers of children and adolescents without diabetes were selected for the non-diabetes group. The invitation to participate in the diabetes group was carried out through the social media of the Juvenile Diabetes Association, a society that integrates associations throughout Brazil meant for children and adolescents with diabetes. The invitation to the non-diabetes group was carried out through social media of medical students across the country. An electronic invitation was sent to access an online survey between May 18th and June 9th of 2020, approximately two months after the beginning of the pandemic in Brazil and after the disclosure from the national ordinance that recommended social distancing for high-risk groups. At the time of the evaluation, Brazil was already considered one of the epicenters of the pandemic. National legislation restricted the functioning of establishments that offer essential services, and which regulated the indication of an exceptional teleworking regime for people with respiratory diseases, immunosuppressed or with chronic disease, upon medical recommendation. Moreover, school activities and non-emergency medical consultations have been suspended. The manuscript description follows the STROBE guideline^12^.

### Participants

Adults of any age who were parents or the primary caregivers of children and adolescents aged less than or equal to 18 years-old with type 1 diabetes were selected for the diabetes group. For the non-diabetes group, adults who were parents or the primary caregivers of children and adolescents aged less than or equal to 18 years-old without diabetes, and who were invited through the social media of student leagues across the country were selected.

### Variables and data source

#### Demographic and clinical data

Sociodemographic information about the caregivers and clinical information about the dependent under their responsibility [such as age, presence of comorbidities, diabetes duration, age at diagnosis, use of medication, last hemoglobin A1c (HbA1c) levels, and presence of diabetes complications] were collected at the beginning of the questionnaire.

#### Support and relationships

After the gathering of initial information, an evaluation about the participant’s social support and relationships was carried out using specific questions (see supplementary material). Participants were asked to choose, based on the last six months (to evaluate periods in addition to the pandemic period), one of the following options regarding the topics already mentioned: “most of the time”, “occasionally” or “almost never/never”. For analysis, a negative response was considered when participants answered “almost never / never”.

#### Consequences of social distancing

Regarding the routine during the pandemic, the questionnaire included “yes”, “no” or “partially” response options for the participants’ compliance regarding social distancing since the beginning of the social distancing recommendation, the time spent by the child at home and possible difficulties related to income and medical care. It was considered that family income decreased if the patient answered yes to the reduction of some source of family income since the beginning of the pandemic.

### Outcomes

The primary outcomes of this study included the presence of pandemic-related emotional burden, the presence of positive screening for mental disorders, and the presence of diabetes-specific emotional burden related to the diabetes care during the period of social distancing, which was caused by the COVID-19 pandemic.

### Pandemic-related emotional burden

The evaluation of pandemic-related emotional burden was carried out for both diabetes and non-diabetes groups and was performed using a 5-point Likert scale for different domains. The domains were evaluated using the following sentences: (1) personal concern—“I often feel worried and afraid of being infected with the coronavirus”; (2) child-related concern—“I often feel worried and afraid that my child may be infected with the coronavirus”; (3) personal emotional burden: “I often feel tired and exhausted due to the changes in routine since the social distancing related to the COVID-19 pandemic started”; (4) child-related emotional burden: “I often feel tired and exhausted from the responsibility to protect my child during the COVID-19 pandemic”. For the analyses, “totally agree” and “agree” were considered affirmative answers.

### Screening for mental health disorders during social distancing

The evaluation of mental health disorders was carried out for both diabetes and non-diabetes groups. For tracking mental health disorders such as anxiety-related disorders, depression and somatoform disorders, the Self-Reporting Questionnaire (SRQ—20) was used in a version previously validated to the Brazilian population^13^. “Yes” or “No” answers were requested for each statement, and a positive screening was considered when the participant answered “Yes” in at least seven of the 20 items^13^_._

### Diabetes-specific emotional burden related to diabetes care

The evaluation of diabetes-specific emotional burden was applied only to the diabetes group using a 5-point Likert scale. The following statements were used for the domains evaluated: (1) care sharing: “I feel frustrated because I am the only one responsible for helping my child in using medications and managing glycemic control”; (2) support: “I feel frustrated with the lack of understanding and support I get from my friends and family in relation to taking care of someone with diabetes”; (3) appreciation: “I feel underestimated for all the effort I put into helping my child to take care of diabetes”; (4) exhaustion: “I feel that my child's diabetes is consuming a lot of my physical and mental energy every day”; (5) guilt: “I feel guilty if my child's diabetes is not well controlled”. In order to facilitate interpretation, answers were categorized in (a) it is a problem, if the answer was “totally agree” or “agree”; (b) it is not a problem, if the answer was “strongly disagree” or “disagree”; and (c) not decided, if the answer was “not decided”.

### Sample size

The Krejcie and Morgan (1970) formula was used to determine sample size for a given population and for an analysis using a 95% confidence level and a margin of error of 0.05^14^. Considering the estimate of 95,800 individuals with diabetes and under 18 years of age in Brazil in 2019, 380 responses in the diabetes group was determined to be necessary to obtain the adequate power for the analyses performed^15^. The strategy was repeated for the non-diabetes group to obtain homogeneity in the procedure performed.

### Statistical methods

The data were transcribed from the online platform SurveyMonkey, (San Mateo, CA, U.S.A.; http://www.surveymonkey.com) to the Statistical Package for Social Science (SPSS®) version 20 for analysis. A hierarchical testing strategy was performed to deal with the problem of multiplicity (*type I error inflation*), and the following outcomes were ranked in descending order of importance: (1) presence of pandemic-related emotional burden, (2) mental health during social distancing and (3) presence of diabetes-specific emotional burden during the COVID-19 pandemic. Just for those outcomes, P values were reported for until the first outcome with a P value greater than 0.05 was obtained and further tests were considered only for exploratory analyses. For all other analyses *(baseline characteristics and psychosocial profile during the pandemic)*, the P value will be reported as usually done. For the diabetes-specific emotional burden related to diabetes care, considering that only the diabetes group was evaluated, P values are not shown.

Descriptive data are presented as mean ± standard deviation (SD) or frequency (%). Statistical analyses of the results include Chi-square tests for categorical variables and *t* tests for continuous parametric variables. Logistic regressions were used to correct for possible confounders and data are presented as odds ratios (OR) and their respective 95% confidence intervals (CI). Only participants who answered at least 75% of the questionnaire were included on the analyses. The missing values are excluded from the analysis if the participant did not answer the question (listwise deletion). A sensitivity analysis was performed to assess psychosocial aspects in caregivers based on different age groups [preschoolers (< 6 years), young children (between 6 and 12 years), and adolescents (> 12 years)], and the results were adjusted for the youth's age, ethnicity, region of origin and income using multivariable logistic regressions. Moreover, considering that there was a high prevalence of chronic diseases in the non-diabetes group, a sensitivity analysis with a healthy control group was performed, including only the participants in the non-diabetes group who did not report chronic diseases. Finally, subgroup analyses are presented as OR and their respective 95% CI and represent the likelihood of subgroups of interest present diabetes-specific emotional burden. An alpha value < 0.05 was used to determine statistical significance.

### Ethics approval and consent participate

The study was done in accordance with the Helsinki Declaration, 2004, and performed in accordance with all relevant guidelines and regulations. The project was approved by the research ethics committee of the main researcher's institution (number 4.045.411). All patients included in the study agreed to the informed consent form available on the online platform before completing the questionnaire.

## Results

### Characteristics of the participants

A total of 1011 responses to the online questionnaire were collected: 485 from those responsible for children and adolescents with diabetes (diabetes group) and 526 from those responsible for children and adolescents without diabetes (non-diabetes group). After excluding participants who did not agree with the informed consent, did not meet the inclusion criteria or completed less than 75% of the questionnaire, 381 participants in the diabetes group and 383 in the non-diabetes group were included in the analyses (see supplementary Fig. 1).

Overall, the participants included in the study had a mean age of 39.9 ± 8.5 years; 95.2% were female, 78.3% were white and 47.8% had medium–low family income. Regarding the relationship with the child, 89.1% were mothers, either biological or adoptive. The diabetes and non-diabetes groups did not differ in relation to the participants' age, sex, and relationship with the child. There was a higher prevalence of non-white and low-income participants in the diabetes group (31.2% vs. 12.3%, *P* < 0.001 and 54.1% vs. 41.5%, *P* = 0.001, respectively) (see Table 1). Representatives from all regions of Brazil were included, although a high proportion of participants in both groups were from the South-Southeast regions (79.0% in diabetes group and 97.4% in non-diabetes group).

**Table 1:** Demographics and clinical characteristics of study participants.

	Total (n = 764)	Non-diabetes group (n = 383)	Diabetes group (n = 381)	*P value*
Age (years)	39.9 ± 8.5	39.5 ± 9.0	40.3 ± 8.0	0.201
Sex (% female)	95.2%	95.0%	95.3%	0.879
Race/ethnicity (% white)	78.3%	87.7%	68.8%	< 0.001
Lower-middle income* (%)	47.8%	41.5%	54.1%	0.001
Parentage (% mother)	89.1%	88.5%	89.8%	0.166
Age of the child (years)	10.0 ± 4.9	8.1 ± 4.7	11.8 ± 4.3	< 0.001
Chronic illness in the child (%)	64.1%	28.5%	100%	< 0.001
Age of the child at diagnosis (years)	6.0 ± 4.4**	2.9 ± 3.4**	6.9 ± 4.3	< 0.001
Disease duration (years)	5.1 ± 4.0**	5.7 ± 4.3**	5.0 ± 3.8	0.096
Continuous-use medication (%)	57.5%	15.1%	100%	< 0.001

Data are mean ± standard deviation or %. α ≤ 0.05 indicates significant difference. *Family that receives a total of less than 2564 reais per month, as defined by the *Strategic Affairs Secretariat (SAE)* of Brazil in 2012, equivalent to 495.8 dollars or 430 euros. **Data included only participants in the non-diabetes group who have some chronic disease.

Children and adolescents in the diabetes group were older (11.8 ± 4.3 vs. 8.1 ± 4.7 years, *P* < 0.001); 28.5% of the participants in the non-diabetes group reported the presence of a chronic illness in the child. Among these participants, 83 had respiratory tract diseases (asthma, bronchitis, cystic fibrosis or rhinitis requiring continuous treatment), 3 had congenital heart disease, 7 had neuro-psychiatric diseases (autism spectrum disorder, attention deficit and hyperactivity or anxiety in need of pharmacological treatment), 1 had epilepsy, 1 polycystic kidney disease, 1 lactose intolerance, 1 deafness and 1 cerebral palsy. Of these, 15.1% used medication daily.

### Support, relationships, and consequences of social distancing

Considering the social-demographic differences between the two groups, the OR for the characteristics evaluated in the psychosocial profile were adjusted for age of the child, race/ethnicity, income, and region of origin. Participants in the diabetes group more frequently reported unsatisfactory relationships (18.0% vs. 9.4%, adjusted OR 1.90; 95% CI, 1.16 to 3.12) and regarding family atmosphere, the diabetes group more frequently reported the atmosphere as not-welcoming (8.0% vs. 1.3%, adjusted OR 5.85; 95% CI, 2.09 to 16.43). In addition, a greater purchasing difficulty (38.7% vs. 10.8%, adjusted OR 4.65; 95% CI, 2.93 to 7.38) and a greater difficulty in getting medical assistance when necessary during the pandemic (44.2% vs. 18.5%, adjusted OR 3.36; 95% CI, 2.27 to 4.97) were reported by the diabetes group compared to non-diabetes group. Aspects related to the social distancing, the child being at home full time and reduced family income during the pandemic did not differ between groups (see Table 2). When the evaluation was carried out comparing only to healthy controls, there was no difference between the groups regarding the maintenance of unsatisfactory relationships, but the diabetes group more frequently reported the family atmosphere as not-welcoming (8.0% vs. 0.4%, adjusted OR 17.39; 95% CI, 2.24 to 134.99). There were few differences in the other parameters (see supplementary Table 2).

**Table 2:** Support, relationships, and consequences of social distancing in study participants.

	Non-diabetes group (N = 377) (%)	Diabetes group (N = 381) (%)	OR (95% CI)
Unsatisfactory relationships^c^	9.4	18.0	1.90 (1.16–3.12)
Family atmosphere not-welcoming^c^	1.3	8.0	5.85 (2.09–16.43)
Follows social distancing^a,d^	93.7	97.0	1.60 (0.71–3.65)
Child full time at home^a,d^	79.9	86.1	1.39 (0.89–2.19)
Family income decreased^a,e^	71.4	72.4	0.97 (0.66–1.42)
Purchase difficulty^a,e^	10.8	38.7	4.65 (2.93–7.38)
Difficulty in medical assistance^a,e^	18.5	44.2	3.36 (2.27–4.97)
Positive screening for mental health disorders^b,f^	50.3	69.0	2.43 (1.70–3.47)

Data are prevalence (%) and odds ratio (OR) with confidence interval (95%). An OR greater than 1 means that there was an increase in likelihood to present the psychosocial characteristic evaluated in the diabetes group in relation to the non-diabetes group (OR for comparator = 1). All OR are adjusted for age of the child, race/ethnicity, income and region of origin. ^a^In relation to the period after the beginning of the COVID-19 pandemic. ^b^Positive screening for mental health disorders accessed by a score greater than or equal to 7 on the SRQ-20. ^c^n = 383 for non-diabetes group and n = 377 for diabetes group. ^d^n = 383 for non-diabetes group and n = 331 for diabetes group. ^e^n = 378 for non diabetes group and n = 326 for diabetes group. ^f^n = 374 for non-diabetes group and n = 323 for diabetes group.

### Pandemic-related emotional burden

**Figure 1:**
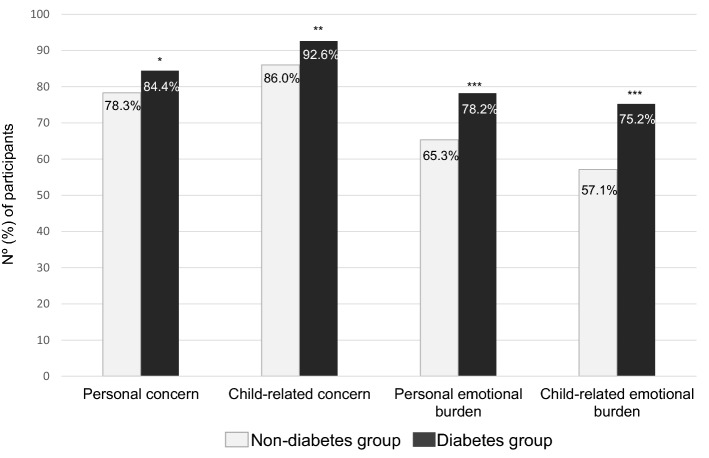
Assessment of pandemic-related emotional burden between the diabetes group and the non-diabetes group.

The emotional burden evaluation during the COVID-19 pandemic was performed in four domains. Participants in the diabetes group most often expressed personal concern (84.4% vs. 78.3%, *P* = 0.041), child-related concern (92.6% vs 86.0%, *P* = 0.005), personal emotional burden (78.2% vs 65.3%, *P* < 0.001) and child-related emotional burden (75.2% vs. 57.1%, *P* < 0.001) when compared to the non-diabetes group (see Fig. 1).

The evaluation of pandemic-related emotional burden between the diabetes group and the non-diabetes group was performed in four domains using the Likert scale of 5 points of agreement. The domains were evaluated through the statements: (1) personal concern—“I often feel worried and afraid of being infected with the coronavirus”; (2) concern related to the child—“I often feel worried and afraid that my child may be infected with the coronavirus”; (3) personal emotional burden: “I often feel tired and exhausted due to the change in routine since the social distancing started due to the COVID-19 pandemic”; (4) child-related emotional burden: “I often feel tired and exhausted from the responsibility to protect my child during the COVID-19 pandemic”. It was considered “totally agree” and “agree” as an affirmative answer.The graphs show the percentages of people with affirmative answers to the proposed statements in each group. *P = 0.041; **P = 0.005; ***P < 0.001.

When adjusted by the age of the child, race/ethnicity, region of origin and income, the diabetes group maintained a greater likelihood of personal concern (OR 1.62; 95% CI, 1.01 to 2.58), a greater likelihood of concern for the child (OR 2.01; 95% CI, 1.11 to 3.64), a greater likelihood of personal emotional burden (OR 1.67; 95% CI, 1.10 to 2.53) and a greater likelihood of burden related to childcare (OR 2.28; 95% CI, 1.54 to 3.38).

A sensitivity analysis, adjusted by the same factors and excluding caregivers of youth with chronic disease in the non-diabetes group, was performed. The diabetes group maintained a greater likelihood of personal concern (OR 1.64; 95% CI, 1.02 to 2.64), a greater likelihood of concern for the child (OR 2.49; 95% CI, 1.35 to 4.59), a greater likelihood of personal emotional burden (OR 2.15; 95% CI, 1.41 to 3.27) and a greater likelihood of burden related to childcare (OR 2.62; 95% CI, 1.74 to 3.92) (see Supplementary Fig. 2).

### Mental health during social distancing

The presence of a positive screening for mental health disorders was found in 50.3% of the participants in the non-diabetes group and in 69.0% of the participants in the diabetes group (*P* < 0.001 for the difference between groups). When adjusted for the age of the child, race/ethnicity, income and region of origin, the diabetes group was found to have a significantly higher likelihood of positive screening for mental health disorders when compared to the non-diabetes group (OR 2.43; 95% CI, 1.70 to 3.47).

A sensitivity analysis, adjusted by the same factors and excluding caregivers of youth with chronic disease in the non-diabetes group, was performed. The diabetes group maintained a significantly higher likelihood of positive screening for mental health disorders when compared to the healthy control group (OR 2.68; 95% CI, 1.82 to 3.96) (see Supplementary Table 2).

### Diabetes-specific emotional burden related to diabetes care

**Figure 2:**
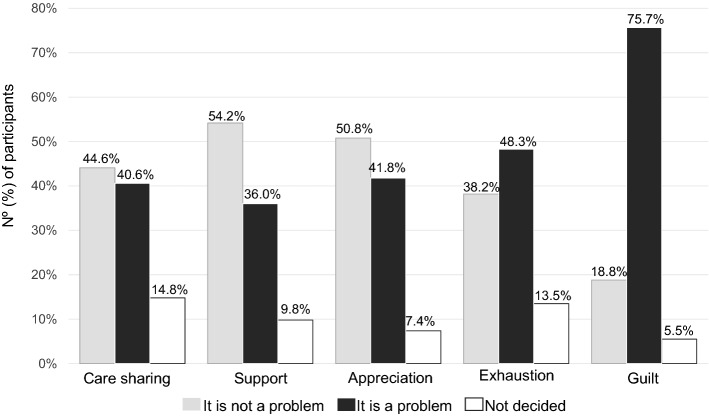
Assessment of diabetes-specific emotional burden related to diabetes care in caregivers of children and adolescents with a previous diagnosis of type 1 diabetes during the COVID-19 pandemic.

In the diabetes group, the diabetes-specific emotional burden related to the care of a child/adolescent with diabetes was evaluated in five domains: 40.6% reported discontent in care sharing, 36.0% reported discontent in support, 41.8% reported discontent in appreciation, 48.3% reported exhaustion and 75.7% reported guilt problems (see Fig. 2).

The evaluation of diabetes-specific emotional burden related to diabetes care was performed using the Likert scale of 5 points of agreement. The domains were evaluated through the statements: (1) sharing of care: “I feel frustrated because I am the only one responsible for helping my child use medications and manage glycemic control.”; (2) support: “I feel frustrated with the lack of understanding and support I get from friends and family in relation to taking care of someone with diabetes.”; (3) appreciation: “I feel underestimated for all the effort I put into helping my child to take care of diabetes.”; (4) exhaustion: “I feel that my child's diabetes is consuming a lot of my physical and mental energy every day.”; (5) guilt: "I feel guilty if my child's diabetes is not well controlled." It was considered that *it is a problem* if the answer was “totally agree” and “agree”, and *it is not a problem* if the answer was "strongly disagree" or "disagree".

An exploratory analysis of subgroups was performed to identify the likelihood of emotional burden related to care of a child/adolescent with diabetes in certain interest groups. The presence of unsatisfactory relationships and family environment that is not welcoming were predictors of having a greater likelihood of reporting emotional burden related to care sharing, support, appreciation, and exhaustion in diabetes care, while the presence of a positive screening for mental health disorders was a predictor for burden in all areas of diabetes care evaluated (see Table 3).

**Table 3:** Subgroup analysis to assess predictors of risk of diabetes-specific emotional burden during the COVID-19 pandemic among guardians of children and adolescents with type 1 diabetes.

Subgroup	Care sharing	Support	Appreciation	Exhaustion	Guilt
Age (participant) > 30 years old	0.98 (0.91–1.05)	0.97 (0.90–1.05)	0.94 (0.88–1.01)	1.01 (0.94–1.08)	0.99 (0.91–1.06)
Age (child) > 8 years old	1.00 (0.89–1.13)	0.95 (0.84–1.09)	1.02 (0.90–1.15)	0.98 (0.87–1.11)	**0.87 (0.77–0.99)**
Race/ethnicity not white	0.75 (0.47–1.21)	0.76 (0.47–1.24)	0.76 (0.47–1.23)	0.78 (0.49–1.26)	1.69 (0.99–2.88)
Diabetes duration > 5 years	1.17 (0.89–1.54)	1.06 (0.80–1.40)	1.19 (0.90–1.56)	1.04 (0.79–1.36)	0.87 (0.64–1.17)
HbA1c > 7.5%	**1.22 (1.02–1.47)**	1.16 (0.97–1.40)	**1.21 (1.01–1.45)**	0.96 (0.80–1.15)	1.00 (0.80–1.24)
Quality of relationships unsatisfactory	**2.07 (1.29–3.33)**	**3.38 (2.07–5.52)**	**2.64 (1.61–4.33)**	**1.63 (1.01–2.63)**	1.11 (0.63–1.95)
Family atmosphere not welcoming	**3.76 (1.62–8.75)**	**13.04 (3.99–42.63)**	**10.19 (3.11–33.37)**	**2.75 (1.18–6.41)**	1.69 (0.60–4.76)
Child full time at home	0.96 (0.88–1.05)	0.99 (0.90–1.08)	1.00 (0.91–1.09)	1.00 (0.91–1.09)	1.02 (0.92–1.14)
Purchase conditions reduced	**1.39 (1.06–1.83)**	**1.49 (1.14–1.95)**	**1.71 (1.30–2.26)**	1.28 (0.97–1.68)	0.86 (0.64–1.16)
Positive screening for mental health disorders	**1.48 (1.29–1.70)**	**1.43 (1.25–1.63)**	**1.44 (1.25–1.66)**	**1.58 (1.36–1.85)**	**1.60 (1.25–2.04)**

Data are odds ratio (OR) and 95% CI and represent the likelihood of subgroups of interest present diabetes-specific emotional burden in the respective domains related to the diabetes care. For each subgroup, the OR was calculated for the subgroup of interest versus the opposite group (eg, family atmosphere not welcoming vs. welcoming). Only participants of diabetes group were included. In bold, the subgroups that presented significantly higher (> 1.00) or lower (< 1.00) likelihood for presenting diabetes-specific emotional burden in the respective domain in relation to the opposite group.

### The impact of the pandemic on different age groups

For the assessment of psychosocial profile and consequences of social distancing on different age groups, the results were adjusted for age of the child, race/ethnicity, income, and region of origin. When considering the different age range, the group of caregivers of youth aged 6 to 12 years with type 1 diabetes most often had unsatisfactory relationships (OR 7.86; 95% CI, 3.59 to 17.22) and had a not-welcoming family atmosphere (OR 5.98; 95% CI, 1.27 to 28.06) in comparison to the non-diabetes group. There was no difference in these aspects in the other age groups. In addition, in all age ranges, caregivers of youth with diabetes had a greater likelihood of having difficulty purchasing during the pandemic. In those aged between 6 and 12 years and those older than 12 years there was a greater likelihood of presenting greater difficulty in getting medical assistance when necessary (OR 3.84; 95% CI, 2.12 to 6.94 and OR 3.69; 95% CI, 1.76 to 7.71, respectively) (see supplementary Table 3).

The emotional burden evaluation on different age ranges was also performed in four domains and adjusted for confounders. The two groups were similar in all domains when the dependent youth was younger than 6 years. On the other hand, there was an increase in likelihood to present child-related concerns (OR 3.19; 95% CI, 1.23 to 8.27 and OR 3.26; 95% CI, 1.22 to 8.71), personal emotional burden (OR 2.05; 95% CI, 1.17 to 3.58 and OR 2.61; 95% CI, 1.34 to 5.10) and child-related emotional burden (OR 2.79; 95% CI, 1.62 to 4.82 and OR 2.56; 95% CI, 1.35 to 4.82) among caregivers of youth with type 1 diabetes aged 6 to 12 years and older than 12 years, respectively (see supplementary Fig. 3).

Regarding mental health disorders, the diabetes group showed greater likelihood of presenting disorders in those aged under 6 years old (OR 2.92; 95% CI, 1.08 to 7.94) and in those aged between 6 and 12 years old (OR 2.89; 95% CI, 1.73 to 4.84). There was no difference between groups in caregivers of adolescents older than 12 years (see supplementary Table 3).

## Discussion

Caring for the mental well-being of people responsible for the care of children and adolescents with diabetes has been undervalued. This study was the first to evaluate the psychological impact of routine changes and disease demands on caregivers of children and adolescents with diabetes during the COVID-19 pandemic. When compared to those responsible for children without diabetes, caregivers of children and adolescents with diabetes more often expressed feelings of concern and burden during social distancing. These participants, even when adjusted for potential confounders, had a 60% higher likelihood of presenting feelings of personal concern and burden and up to twice the likelihood of having feelings of concern and burden related to caring for their child during the pandemic. The care of children and adolescents with diabetes has also been associated with a higher likelihood of having a positive screening for mental health disorders during the social distancing period. Moreover, considering the diabetes group, about 3 out of 4 participants reported feeling guilty when the glycemic control of the dependents was not adequate, almost half reported experiencing feelings of exhaustion related to the care of these children and more than a third reported discontent in sharing care, support from family members and appreciation for their dedicated efforts.

The presence of concern, stress and anxiety in relation to glycemic control are part of the routine of the most caregivers of children with diabetes, and behavioral changes during the COVID-19 pandemic seem to enhance this situation^16^. Participants in the diabetes group more frequently reported the presence of feelings of concern and emotional burden, both personal and related to child care. The longer time at home related to the suspension of non-essential activities may result in an increased demand for those responsible for children with diabetes. Moreover, these individuals are required to coordinate insulin administration and the maintenance of a balanced diet full time.

In our study, those responsible for children and adolescents with diabetes had a higher likelihood of a positive screening for mental health disorders during the social distancing period compared to the non-diabetes group. Previous work has shown that the prevalence of symptoms of depression in the parents of children with newly diagnosed diabetes can reach 74% and, in about 20% of these parents, the symptoms remain for four years after diagnosis^6,17^. The presence of a potentially fatal pandemic may work as a trigger for the manifestation of latent anxiety and depression symptoms. Caregivers with depressive symptoms may perceive the complexities of diabetes and the challenges associated with its treatment as more negative and distressing, and may lack the necessary coping skills to manage this distress^18–20^. In addition, studies have found depressive symptoms in parents are associated with lower parental involvement, lower family adaptability and higher family conflict, which corroborates the relationships found in the present study^6,21–23^.

Regarding diabetes care during the pandemic, those responsible for this group tended to have a higher prevalence of diabetes-specific emotional burden in different domains related to the demands of the disease. Whittemore et al*.* found, in a review of studies focused on parents of children with diabetes in usual situations, that those individuals experience considerable stress related to the demands of treatment management. They found that the prevalence of parental psychological distress ranged from 20 to 30% in different studies and, in most of these studies, parents of children with diabetes were found to experience greater distress and problems with parenting than other parents, which may negatively affect their children^6^. It is difficult to say, based on our study data, how much of the burden found is due to the pandemic and how much is due to the basic conditions of the family members, which may already be weakened. It is possible to imagine, however, that the increase in demands related to social distancing and the anxiogenic context experienced may have contributed to the high levels of diabetes-specific emotional burden reported.

In our study, we chose to use the SRQ-20 questionnaire to assess the prevalence of mental health disorders in caregivers. The choice of this questionnaire was based especially on the wide range of psychiatric disorders assessed (minor disorders) compared to other mental health scores. Other commonly used options, such as the Hospital Anxiety and Depression Scale (HADS) and the Patient Health Questionnaire-9 (PHQ 9), could not be used^24,25^. The first because its validation for the Brazilian population was performed in hospitalized patients and the second because the evaluation is restricted to depressive disorders alone. All of them have been validated for self-application.

It is necessary to highlight some limitations of the present study. Considering that it is a cross-sectional study, it is not possible to establish cause and effect relationships using the identified associations; reverse causality may occur. The unavailability of validated and translated questionnaires to evaluate anxiety diabetes-specific distress in caregivers has limited the use of these tools in our population. In addition, given that this was an online survey, the results depend on the commitment of the participants and on the veracity of the information provided, which may constitute potential information bias. The dissemination of the questionnaire to the non-diabetes group through medical student leagues may also have selected families with the sickest children in this group and may not be a representative sample of the general population. Regarding the participants sample, although both questionnaires were released at the national level, the diabetes group had a greater representation of different regions of the country. Most participants are from the South and Southeast regions of the country, which limits the generalization of the results. The non-diabetes group also showed less ethnic diversity and greater purchasing power, which may be related to the greater representation of states in Southern Brazil. Although all analyses have been adjusted for the region of origin, purchasing power, youth’s age and ethnicity, these differences in baseline characteristics between groups may reflect a sample bias related to the study design and constitute a limitation of the manuscript. Adjusted analyses for age of the child, skin color, income and region of origin were performed to minimize the effect of these differences. Another aspect that should be highlighted is the high representativeness of female caregivers, which can reduce the validity of the results for male representatives. This high prevalence is in line with other studies conducted in pediatric outpatient care in Brazil, in which the mother plays the role of primary caregiver in 91.0 to 93.7% of cases^26,27^. Finally, it is necessary to highlight the possibility of selection bias among the participants because family members who are more engaged in childcare may be more interested in participating in the research.

Despite the limitations presented, our study should call attention to mental health and to the diabetes-specific emotional burden related to diabetes care during the pandemic. The evidence found suggests that situations of vulnerability and public calamity may enhance symptoms of concern, burden and mental health disorders in some caregivers of children and adolescents with diabetes, and studies comparing the time periods prior to and after the pandemic should be carried out to better understand this relationship. These results also reveal that additional studies are necessary to understand the burden that these families may be experiencing during the current scenario and to develop new strategies to support these caregivers, especially during the pandemic. The "caregivers and children" binomial approach is fundamental for treating the child's diabetes, and for taking care of the well-being and quality of life of everyone involved.

## Supplementary Information

Supplementary Information

## Data Availability

The data collected for the study, including deidentified participant data, will be available for 1 year after publication of the article upon justified request to the e-mail address of the main researcher and with a signed data access agreement.
