# Supplementary material for: Caring for caregivers: the impact of the COVID-19 pandemic on those responsible for children and adolescents with type 1 diabetes
Source: Sci Rep. 2021 Mar 24;11:6812. doi: 10.1038/s41598-021-85874-3 (PMC7991637; doi:10.1038/s41598-021-85874-3)
Supplement: Supplementary file 1 — Supplementary Information [file 41598_2021_85874_MOESM1_ESM.pdf]

SUPPLEMENTARY MATERIAL

Caring for caregivers: the impact of the COVID-19 pandemic on those responsible for children and adolescents with type 1 diabetes.

Running title: COVID-19 pandemic and caregivers of children with diabetes

Authors: Janine Alessi, MD[1,2]; Giovana Berger de Oliveira[3]; Gabriela Feiden, BSEd[4,5]; Beatriz D. Schaan, MD PhD[1,6,7,8]; Gabriela Heiden Telo, MD PhD[2,3,8,9]

1. Graduate program in Medical Science: Endocrinology, Universidade Federal do Rio Grande do Sul, Brazil
2. Internal Medicine department, Hospital São Lucas - Pontifícia Universidade Católica do Rio Grande do Sul, Brazil
3. School of Medicine, Pontifícia Universidade Católica do Rio Grande do Sul, Brazil
4. Graduate program in Epidemiology, Universidade Federal do Rio Grande do Sul, Brazil
5. Associação de Apoio aos Diabéticos do Rio Grande do Sul (AADIRS), Brazil
6. School of Medicine, Universidade Federal do Rio Grande do Sul, Brazil
7. Endocrinology division, Hospital de Clínicas de Porto Alegre, Brazil
8. National Institute of Science and Technology for Health Technology Assessment (IATS) – CNPq/Brazil
9. Graduate program in Medicine and Health Sciences, Pontifícia Universidade Católica do Rio Grande do Sul, Brazil

Corresponding Author: Janine Alessi

E-mail: janinealessi@gmail.com

Address: Hospital de Clínicas de Porto Alegre

Rua Ramiro Barcelos, 2350, prédio 12, 4º andar

90035-003 - Porto Alegre, RS, Brasil

Phone: +55-51-3359.8127

Methods:

1. Support and relationships

Participants were asked to answer the following questions:

1) Satisfactory relationships: "Do you consider that you have satisfactory relationships (i.e, do you feel that you can share good times, as well as you can count on friends or family when you have difficulties and needs?)”.

2) Welcoming family environment: "Do you consider your family environment welcoming (that is, people are affectionate and loving? Do you feel comfortable in your daily lives)?".

Participants were asked to choose based on the last six months one of the following options regarding the topics already mentioned: “most of the time”, “occasionally” or “almost never / never”. For analysis, a negative response was considered when participants answered “almost never / never”.

2. Consequences of social distancing

It was considered that family income decreased if the patient answered yes to the reduction of some source of family income since the beginning of the pandemic.

Purchase difficulty was considered if the participant answered "yes" to the question: "Did you have any financial difficulties to buy food or medicine during the pandemic?".

Difficulty in medical care was considered if the participant answered "yes" to the question: "Did you have any difficulty getting medical care for your child at any time that you considered necessary?”.

Supplementary table 1. Demographics and clinical characteristics of study participants considering only the healthy control group

	**Total** (n = 655)	**Healthy control group** (n = 274)	**Diabetes group** (n = 381)	***P value***
**Age (years)**	40.1 ± 8.7	40.0 ± 9.6	40.3 ± 8.0	0.535
**Sex (% female)**	94.5%	93.4%	95.3%	0.301
**Race/ethnicity (% white)**	77.4%	89.4%	68.8%	<0.001
**Lower-middle income* (%)**	47.0%	37.2%	54.1%	<0.001
**Parentage (% mother)**	87.9%	85.4%	89.8%	0.069
**Age of the child (years)**	10.2 ± 4.9	8.0 ± 4.7	11.8 ± 4.3	<0.001
**Chronic illness in the child (%)**		-	100%	
**Age of the child at diagnosis (years)**		-	6.9 ± 4.3	
**Disease duration (years)**		-	5.0 ± 3.8	
**Continuous-use medication (%)**		-	100%	

Data are mean ± standard deviation or %. α ≤ 0.05 indicates significant difference. *Lower-middle income: family that receives a total of less than 2564 reais per month, as defined by the *Strategic Affairs Secretariat (SAE)* of Brazil in 2012, equivalent to 495.8 dollars or 430 euros.

Supplementary table 2: Support, relationships and consequences of social distancing in study participants, considering only the healthy control group.

	Control group (n = 381)	Diabetes group (n = 381)	OR (95% IC)
Unsatisfactory relationships	9.1%	18.0%	1.74 (0.99 – 3.05)
Family atmosphere not-welcoming	0.4%	8.0%	17.39 (2.24 – 134.99)
Follows social distancing	93.4%	97.0%	0.68 (0.29 – 1.62)
Child full time at home	79.6%	86.1%	1.45 (0.89 – 2.38)
Family income decreased	68.3%	72.4%	1.02 (0.67 – 1.54)
Purchase difficulty	9.6%	38.7%	4.89 (2.87 – 8.32)
Difficulty in medical assistance	15.1%	44.2%	4.00 (2.54 – 6.30)
Positive screening for mental health disorders†	46.6%	69.0%	2.68 (1.82 – 3.96)

Data are prevalence (%) and odds ratio (OR) with confidence interval (95%). An OR greater than 1 means that there was an increase in likelihood to present the psychosocial characteristic evaluated in the diabetes group in relation to the comparator group (OR for control group = 1). All odds ratio are adjusted for age of the child, race/ethnicity, income and region of origin. † Positive screening for mental health disorders accessed by a score greater than or equal to 7 on the SRQ-20.

Supplementary table 3: Support, relationships and consequences of social distancing in study participants considering age range of the dependent.

	**Diabetes group OR (95% IC)**
Age < 6 years	n = 34
Unsatisfactory relationships	1.93 (0.68 – 5.47)
Family atmosphere not-welcoming	2.83 (0.43 – 18.86)
Purchase difficulty	6.50 (2.37 – 17.81)
Difficulty in medical assistance	1.88 (0.75 – 4.75)
Positive screening for mental health disorders †	2.92 (1.08 – 7.94)
Age ≥ 6 and ≤ 12 years	n = 144
Unsatisfactory relationships	2.37 (1.14 – 4.93)
Family atmosphere not-welcoming	5.98 (1.27 – 28.06)
Purchase difficulty	7.86 (3.59 – 17.22)
Difficulty in medical assistance	3.84 (2.12 – 6.94)
Positive screening for mental health disorders †	2.89 (1.73 – 4.84)
Age > 12 years	n = 146
Unsatisfactory relationships	1.29 (0.49 – 3.35)
Family atmosphere not-welcoming	*
Purchase difficulty	2.49 (1.12 – 5.52)
Difficulty in medical assistance	3.69 (1.76 – 7.71)
Positive screening for mental health disorders †	1.83 (0.99 – 3.38)

Data are odds ratio (OR) with confidence interval (95%). %). An OR greater than 1 means that there was an increase in likelihood to present the psychosocial characteristic evaluated in the diabetes group in relation to the non-diabetes group (OR for non-diabetes group = 1). All odds ratio are adjusted for age of the

child, race/ethnicity, income and region of origin. For age < 6 years, n = 150 in the non-diabetes group. For age ≥ 6 and ≤ 12 years, n = 150 in the non-diabetes group. For age > 12 years, n = 74 in the non-diabetes group. *There is not enough data for the analysis. † Positive screening for mental health disorders accessed by a score greater than or equal to 7 on the SRQ-20.

Supplementary table 4: Support, relationships and consequences of social distancing in study participants considering ethnicity.

	**Diabetes group OR (95% CI)**
Race/ethnicity white	n = 262
Unsatisfactory relationships	1.63 (0.91 – 2.88)
Family atmosphere not-welcoming	4.19 (1.37 – 12.79)
Purchase difficulty	4.46 (2.65 – 7.52)
Difficulty in medical assistance	3.03 (1.95 – 4.71)
Positive screening for mental health disorders †	2.06 (1.39 – 3.06)
Personal concern	1.32 (0.81 – 2.14)
Child-related concern	2.25 (1.20 – 4.24)
Personal emotional burden	2.10 (1.37 – 3.23)
Child-related emotional burden	1.94 (1.31 – 2.90)
Race/ethnicity not white	n = 119
Unsatisfactory relationships	3.65 (1.12 – 11.90)
Family atmosphere not-welcoming	1.05 (0.94 – 1.19)
Purchase difficulty	6.99 (2.30 – 21.21)
Difficulty in medical assistance	5.56 (2.09 – 14.80)
Positive screening for mental health disorders †	4.69 (1.96 – 11.22)
Personal concern	3.21 (0.98 – 10.49)
Child-related concern	2.39 (0.55 – 10.53)
Personal emotional burden	2.19 (0.81 – 5.90)
Child-related emotional burden	6.84 (2.49 – 18.79)

Data are odds ratio (OR) with confidence interval (95%). An OR greater than 1 means that there was an increase in likelihood to present the psychosocial

characteristic evaluated in the diabetes group in relation to the non-diabetes group (OR for non-diabetes group = 1). All odds ratio are adjusted for age of the child, income and region of origin. For race/ ethnicity white, n = 336 in the non-diabetes group. † Positive screening for mental health disorders accessed by a score greater than or equal to 7 on the SRQ-20.

Supplementary figure 1.

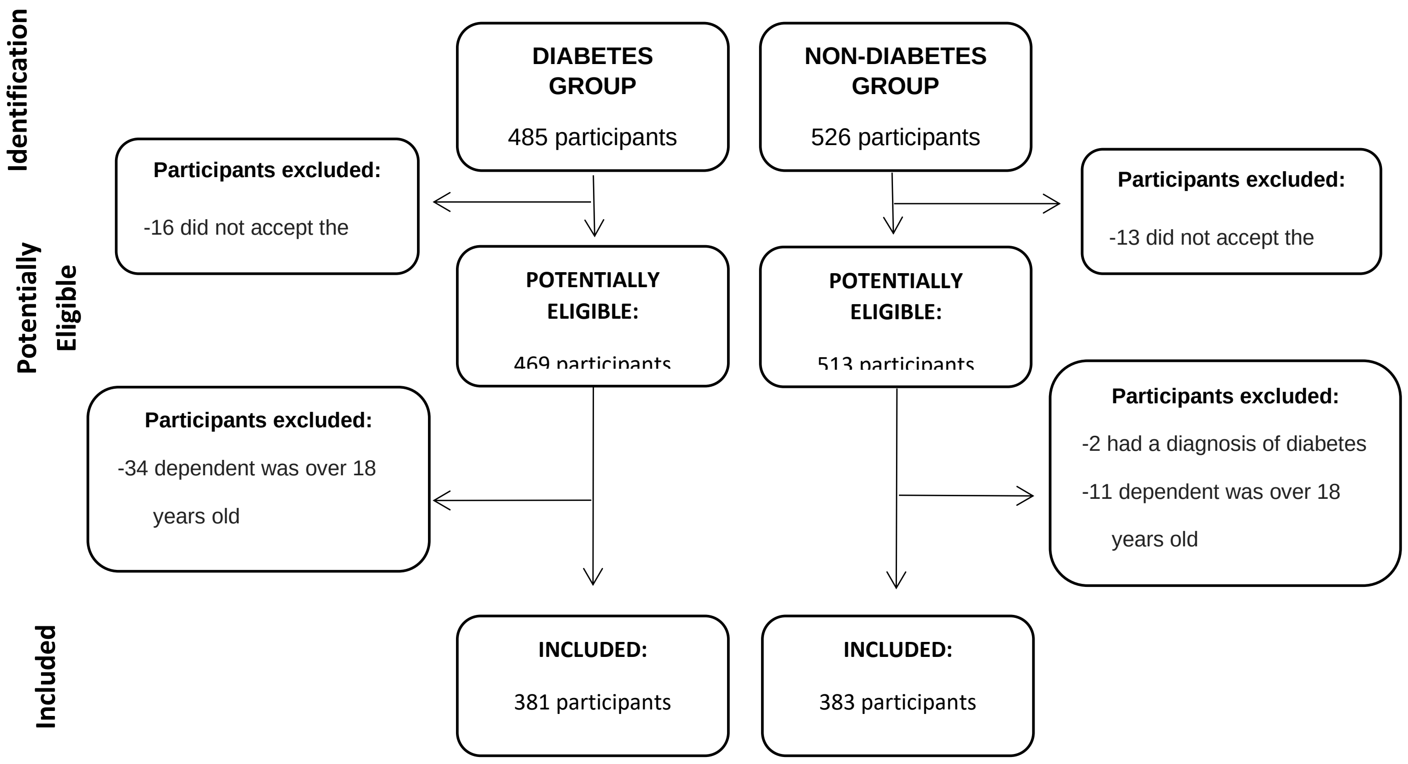

Number of patients identified, eligible and included in the study in the diabetes and non-diabetes groups.

Supplementary figure 2. Assessment of pandemic-related emotional burden between the diabetes group and the healthy control group**.**

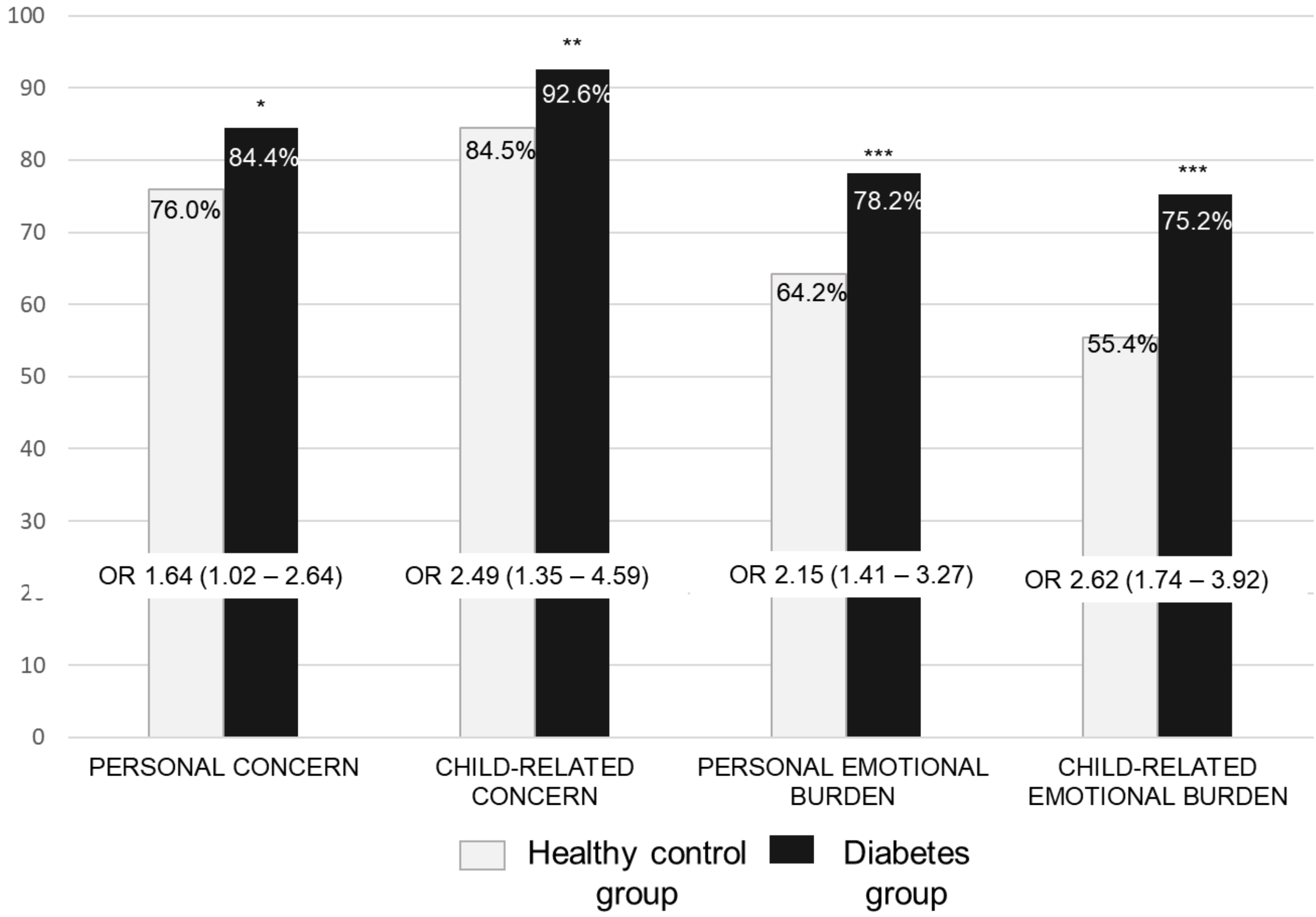

In this evaluation, we included only participants who were caregivers of healthy children, without chronic diseases in the control group (n= 274). The graphs show the percentages of people with affirmative answers to the proposed statements in each group and OR (95% IC) adjusted by the age of the child, race/ethnicity, region of origin and income. *P = 0.01; ** P<0.01; *** P<0.001.

Supplementary figure 3. Assessment of pandemic-related emotional burden between the diabetes group and the non-diabetes group according to the age range of the youth.

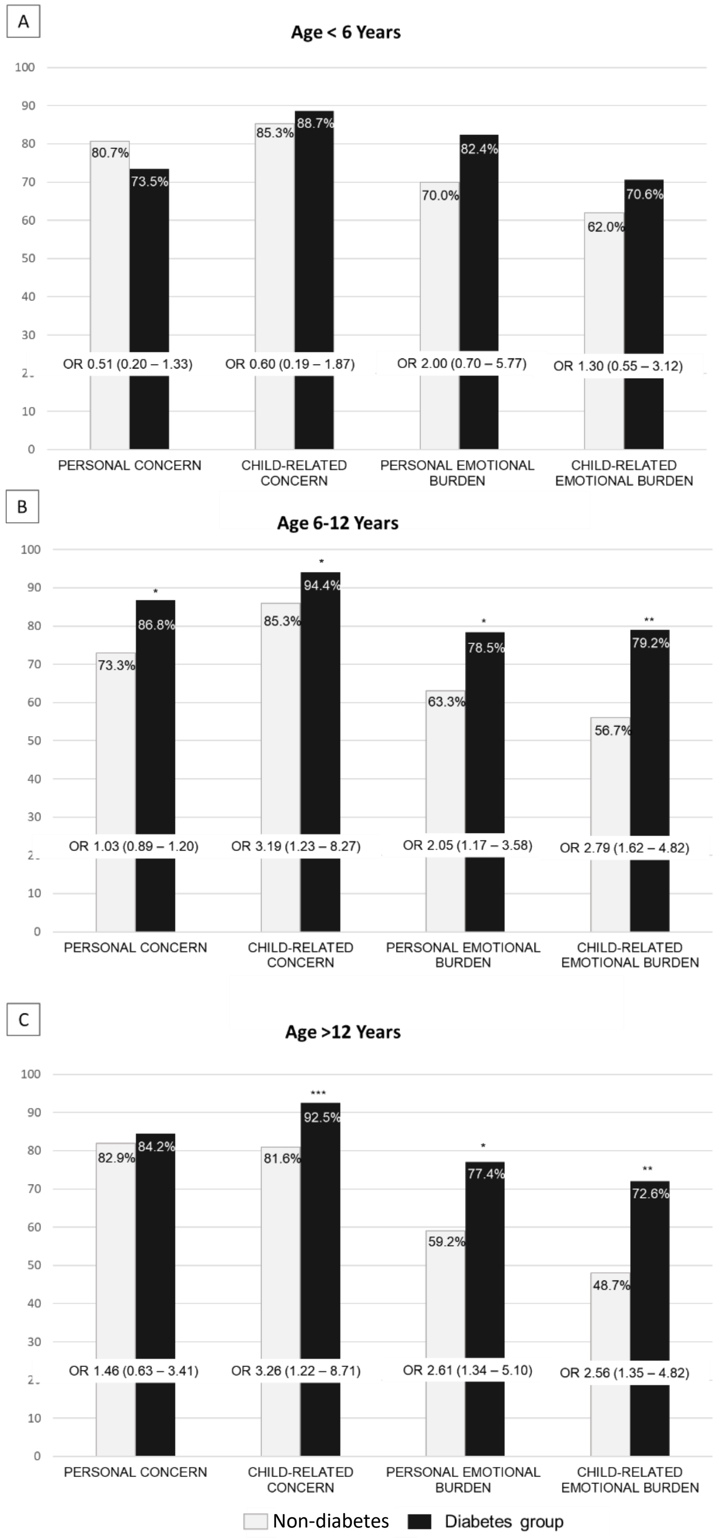

The graphs show the percentages of people with affirmative answers to the proposed statements in each group and OR (95% IC) adjusted by the age of the child, race/ethnicity, region of origin and income. A: Includes children under 6 years old. B: Includes youth aged 6 to 12 years. C: Includes adolescents over 12 years old. * p = <0.01 ** p < 0.001 *** p = 0.02
